# The Role of Endocan in Cardiometabolic Disorders

**DOI:** 10.3390/metabo13050640

**Published:** 2023-05-08

**Authors:** Aleksandra Klisic, Dimitrios Patoulias

**Affiliations:** 1 Primary Health Care Center, Faculty of Medicine, University of Montenegro, 81000 Podgorica, Montenegro; 2 Outpatient Department of Cardiometabolic Medicine, Second Department of Cardiology, Aristotle University of Thessaloniki, General Hospital “Hippokration”, 54642 Thessaloniki, Greece; dipatoulias@gmail.com

**Keywords:** atherosclerosis, endocan, endothelial dysfunction, inflammation

## Abstract

Discovered two decades ago, endocan still represents an intriguing biomarker related to inflammation. Endocan is a soluble dermatan sulphate proteoglycan secreted by endothelial cells. Its expression is observed in tissues related to the enhanced proliferation, especially hepatocytes, lungs, kidneys, etc. Endocan has been investigated in many cardiometabolic disorders that are tightly connected with inflammation, such as type 2 diabetes mellitus, hypertension, atherosclerotic cardiovascular disease, kidney disease, obesity, polycystic ovary syndrome, metabolic syndrome, non-alcoholic fatty liver disease, etc. In this narrative, comprehensive review of the currently available literature, special attention will be paid to the role of endocan in the broad spectrum of cardiometabolic disorders. Since endocan has emerged as a novel endothelial dysfunction marker, the discovery of potential therapeutic strategies for patients with certain cardiometabolic risk factors would be of great importance to delay or even prevent the onset and progression of related complications, mainly cardiovascular.

## 1. Introduction

Endocan (previously called endothelial-specific molecule, ESM-1) is a new proteoglycan of a low molecular weight discovered two decades ago [1]. It is considered an endothelial dysfunction marker since endothelial cells are the key site of its secretion [2].

### 1.1. Structure of Endocan

Unlike many other dermatan sulphate (DS) proteoglycans which are much larger, endocan differs in several ways: (1) it is a DS of low molecular weight (approximately 50 kDa); (2) it is a soluble, secreted DS, but is not linked to the cell membrane or extracellular matrix, which enables endocan to act at distant cells; thus, it can be detected in circulation; (3) it has only one single short DS chain, but not multiple glycosaminoglycan chains, and (4) it has a higher content of disulphated disaccharides and non-sulphated disaccharides, which makes endocan specific for the binding of growth factors with high affinity [1,2].

A gene called ESM-1 is responsible for encoding endocan in humans [1,2]. Endocan consists of a protein core (of 165 amino acids) that exhibits 2 domains, i.e., the N-terminal cysteine-rich (of 110 amino acids) and cysteine-free (of 55 amino acids) domains. The latter is divided into three parts, i.e., the C-terminal region (at the very end of the cysteine-free domain), the phenylalanine-rich region (in the middle part of the cysteine-free domain), and the epidermal growth factor-like region (the closest to the cysteine-rich domain). The core protein is linked to the chain of glycosaminoglycan by serine 137, through post-translational modification.

### 1.2. Secretion and Expression of Endocan

Although endocan is secreted by endothelial cells, its expression is observed in tissues related to enhanced proliferation, especially hepatocytes, bronchial epithelium, cardiomyocytes, neurons, glandular, and kidney tissue [1,2].

Previous studies have shown higher expression of endocan in patients suffering from severe sepsis [2]. The overexpression of endocan is also recorded in cancer cells, indicating that this proteoglycan takes part in the promotion of the growth of tumors, which may partly explain its higher expression in some carcinomas [3].

Stimulation of endocan secretion occurs due to a wide variety of proinflammatory mediators, such as tumor necrosis factor-alpha (TNF-α), interleukin-1, fibroblast growth factor-2, and vascular endothelial growth factor-A (VEGF-A) [1,2]. It has also been shown that in vivo endocan expression is enhanced by several mitogens in adipocytes, such as phorbol ester and retinoic acid [4]. On the other hand, interferon-γ downregulates endocan expression [1,2].

The half-life of endocan is approximately one hour, whereas its catabolism is not clear. It is assumed that neutrophil proteases are responsible for this process, as shown in in vitro studies [2].

### 1.3. Mechanism of Action of Endocan

Endocan plays a significant role even in the early stages of atherosclerosis [1,2,5]. The endothelial dysfunction properties that endocan exhibits include several crucial biologic actions, such as modulation of leukocytes’ migration, leukocytes’ adhesion, and vascular smooth-muscle cell proliferation [2]. Endocan, per se, can also stimulate the endothelium to secrete a variety of cytokines that force the migration of leukocytes [5] and enhance the permeability of blood vessels [5].

The proinflammatory actions of endocan occur via nuclear factor kappa B (NF-κB) and mitogen-activated protein kinase (MAPK) signaling pathways. These processes lead to enhanced expression of cell adhesion molecules (i.e., intercellular adhesion molecule (ICAM-1), vascular cell adhesion molecule (VCAM-1), and E-Selectin), which favors leukocytes’ migration and adhesion to the endothelial cells [2,5].

VEGF-A is a biomarker that stimulates endocan secretion and facilitates the activity of endocan on cell adhesion molecules via binding of VEGF-A to its receptor. VEGF-A enhances endocan mRNA and protein expression in endothelium via the protein kinase C (PKC)/nuclear factor kappa B (NF-κB) pathway, whereas phosphoinositide 3-kinase (PI3K) has an inhibitory effect on this process. VEGF-A favors the permeability of blood vessels [1,2,5].

Endocan has been shown to increase the proliferation of vascular smooth-muscle cells, thus being involved in the formation of neointima, which further contributes to atherogenesis [1,2,5].

Endocan also takes part in neo-angiogenesis by favoring cells’ proliferation, being associated with tumor growth [3,5].

The properties of endocan are briefly summarized in Table 1.

**Figure 1:**
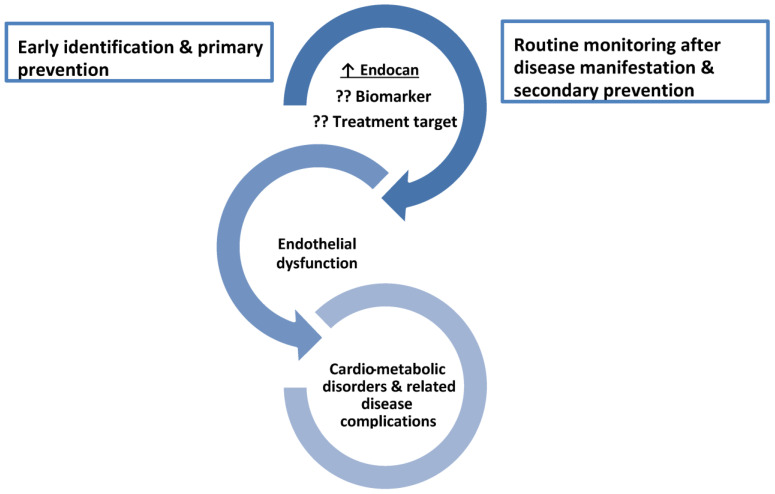
The role of endocan as a biomarker and a treatment target across the spectrum of cardiometabolic disorders. ↑ increased level; ?? it needs to be confirmed due to contradictory results.

In the present narrative review article, special attention will be paid to the role of endocan in cardiometabolic disorders, based on the fact that high levels of this biomarker have been documented in several disorders closely associated with insulin resistance (IR) and inflammation [6,7,8,9,10,11]. Thus, endocan could be a novel treatment target in the spectrum of cardiometabolic disorders (Figure 1).

## 2. Endocan and Obesity

Obesity is a well-established risk factor for cardiometabolic disorders [12,13]. Adipose tissue secretes numerous proinflammatory cytokines and adipokines [6]. Since obesity is considered a state associated with low-grade, persistent, chronic inflammation that leads to atherogenesis onset and atherosclerotic cardiovascular disease (ASCVD) [6,12], it is expected that the serum endocan level (as a proinflammatory mediator) will be increased in obese individuals.

However, the existing data regarding the association between endocan and obesity are controversial. While some studies have shown higher endocan levels in obesity [6] and a positive correlation between anthropometric measurements of both overall obesity (i.e., body mass index (BMI)) [10,14,15] and abdominal obesity (i.e., waist circumference (WC)) [10], others have reported the opposite, i.e., lower endocan levels in obesity [16] and a negative association with the anthropometric indices [17,18,19]. Such controversies might be attributed to the different ethnic groups studied, the phenotypes of adiposity, the duration and extent of obesity, and the sample size of the studied population. Examined groups differed in age and gender distribution, which may also explain these discrepancies [6].

Wellner et al. [4] were the first to investigate endocan’s role in obesity. They showed increased endocan expression by adipocytes and suggested that endocan could take part in regulating the inflammatory response [3]. In line with this, after stimulating proinflammatory mediators in the placenta, in a group of women with obesity and gestational diabetes mellitus (n = 40), an increased endocan expression has been recorded compared to pregnant women (n = 10) with standard glucose tolerance [20].

In a relatively large sample of 177 adults, Klisic et al. [6] showed not only a positive correlation of BMI and WC with serum endocan levels but also its independent relationship with some novel, non-traditional anthropometric indices that better reflect cardiometabolic risk than BMI and WC, such as the cardiometabolic index (CMI) (i.e., it includes the waist-to-height ratio and the triglycerides (TG)/high-density lipoprotein cholesterol (HDL-c) ratio and better reflects visceral adipose tissue than WC) and the body adiposity index (BAI) (i.e., it combines hip circumference and body height and better reflects the subcutaneous adipose tissue than BMI) [21,22]. Similar results have been shown in the pediatric population. Nalbantoglu et al. [23] recorded higher circulating endocan levels in children with obesity (n = 80), as compared to the control group. On the contrary, Janke and al. [16] reported lower circulating endocan levels in overweight/obese postmenopausal women compared to normal-weight ones, but their cohort (n = 70) was smaller than that in the previous study [6]. Janke and colleagues [16] further demonstrated lower endocan expression in preadipocytes than in adipocytes of the subcutaneous abdominal adipose tissue of the examined postmenopausal women. Furthermore, endocan expression in adipose tissue cells was not changed, although its circulating levels were higher after the weight reduction program compared to the baseline values [16].

## 3. Endocan and Polycystic Ovary Syndrome

Polycystic ovary syndrome (PCOS) is a metabolic disorder with IR as the main underlying pathophysiologic mechanism. It usually affects premenopausal women [24], and in addition to metabolic changes (such as central obesity, dyslipidemia, dysglycemia, hypertension), endothelial dysfunction is also observed in PCOS, suggesting that women with PCOS are at increased risk of cardiovascular disease (CVD) [25].

Studies that investigated the relationship between endocan and PCOS are scarce and discrepant results have been reported. In a cross-sectional study that included 88 women of reproductive age with PCOS, and 87 age- and BMI-matched controls, the PCOS group exhibited higher serum endocan levels [19]. However, when classified according to baseline BMI, the normal-weight PCOS women had higher serum endocan levels as compared to overweight/obese PCOS women. Moreover, a positive association between endocan and the HDL-c level, but an inverse association between endocan and BMI and C-reactive protein (CRP), were shown [19]. Similarly, higher serum endocan levels were shown in PCOS women in the study conducted by Bicer et al. compared to the healthy group [10]. This study included a similar sample size (i.e., 80 women in both the PCOS and control groups) as the previously mentioned one [19], but reported a positive association between endocan and BMI, high-sensitivity CRP (hs-CRP), IR (as determined with the homeostasis model assessment of insulin resistance (HOMA-IR)), and the free androgen index (FAI) in both groups. Most importantly, an independent relationship between endocan and carotid intima-media thickness (cIMT) was shown in this study, a finding that implies that this biomarker could be a reliable indicator of a higher risk of CVD in women with PCOS [10].

## 4. Endocan and Metabolic Syndrome

Endothelial dysfunction is one of the first and major pathophysiologic mechanisms implicated in metabolic syndrome (MetS) development, progression, and complication with ASCVD [26]. Iwańczyk et al. [26] showed higher serum endocan levels in individuals with MetS (n = 34), as compared to the MetS-free group (n = 56). Similar results were shown in a pediatric population. More specifically, Halici et al. [27] showed a nearly threefold increase in endocan levels in children with MetS (n = 30) compared to otherwise healthy children (n = 30). Another recent study showed that median circulating endocan values were higher in adults with prediabetes (n = 59) than in the control group (n = 117), although that difference did not reach statistical significance [7]. On the contrary, Arman et al. [28] demonstrated lower levels of circulating endocan in subjects with prediabetes (n = 42) compared to healthy controls (n = 42). Boyuk et al. [29] also recorded lower circulating endocan levels in MetS subjects (n = 44) as compared to MetS-free individuals (n = 26).

Discrepant results were also shown when evaluating the relationship between endocan and each individual component of MetS. Bicer et al. [10] did not find an association between circulating endocan levels and lipid parameters. Delibas et al. [19] recorded a positive correlation between circulating endocan levels and HDL-c, whereas Klisic et al. [30] showed a positive correlation between endocan and TG, as well as a negative association with HDL-c. Moreover, in the latter study [30], diabetes-free subjects with low, small-dense LDL (sdLDL)% and impaired glycoregulation exhibited higher serum endocan levels, as compared to the corresponding group with low sdLDL% and good glycoregulation, which implies a significant influence of IR on higher circulating endocan levels [7]. Moreover, a recent study documented higher serum endocan levels in postprandial lipemia [31], as another independent risk factor for endothelial dysfunction and ASCVD [32].

As for the parameters of glucose homeostasis, Arman et al. [28] showed an inverse association between endocan and fasting insulin levels, whereas a study by Klisic et al. [30] demonstrated a positive association between endocan and fasting glucose and glycated hemoglobin (HbA1c).

## 5. Endocan and Non-Alcoholic Fatty Liver Disease

### 5.1. Circulating Endocan Levels in Non-Alcoholic Fatty Liver Disease

Non-alcoholic fatty liver disease (NAFLD), as a hepatic manifestation of MetS, is characterized by enhanced free fatty acids’ influx in the liver, increments in lipid accumulation, IR, oxidative stress, and inflammation [33,34]. NAFLD has been shown to be an independent cardiovascular risk factor [35]. If persisting, hepatocyte lesions can enhance collagen synthesis and induce hepatocytes’ apoptosis, and further lead to fibrosis, cirrhosis, and hepatocellular carcinoma [36].

Conflicting results have been shown concerning the role of endocan in NAFLD [37,38,39,40,41,42].

Patients with NAFLD and coronary artery disease (CAD) (n = 66) were shown to have higher levels of endocan in circulation compared to individuals with NAFLD who were CAD-free (n = 11) [37]. Endocan levels were shown to be higher in NAFLD (as determined with the fatty liver index) (n = 147), as compared to the corresponding group without liver disease (n = 64) [9]. Additionally, Klisic et al. [9] examined the discriminatory capability of endocan related to NAFLD and found that this biomarker seems to be more reliable in the diagnostic assessment of NAFLD when combined with other biomarkers (e.g., gender, HbA1c, HDL-c, alanine aminotransferase, hsCRP). Higher endocan levels were also recorded in a much smaller sample size study than the previously mentioned one (i.e., n = 32 patients with type 2 diabetes mellitus (T2DM) and NAFLD, as well as n = 19 patients with NAFLD but T2DM-free), as compared to the control group [38]. On the contrary, one earlier study [39] showed lower endocan concentration in subjects with NAFLD (n = 38) than in the controls (n = 34). Similarly, patients with MetS and NAFLD (n = 40) had lower serum endocan levels as compared to the healthy group (n = 20) [40].

As for the children and adolescent population, no difference in circulating endocan levels was shown between obese participants with (n = 40) and without NAFLD (n = 60), as compared to controls (n = 40) [41]. Similarly, there were no differences between serum endocan levels in overweight and obese children (n = 26) with NAFLD and those without (n = 48) [42].

### 5.2. Circulating Endocan Levels in Liver Fibrosis

A recent study [9] reported that the median endocan level in patients with NAFLD (as determined by the fatty liver index) was 38.8 (21.6–89.5) ng/L. The median endocan level in patients with advanced fibrosis (as determined by the BARD score) was 44.2 (22.8–92.7) ng/L, while in controls without liver disease, the median endocan level was 27.8 (17.6–40.9) ng/L, so it is assumed that the increase in serum endocan levels might be associated with the progression of liver disease. Moreover, the diagnostic accuracy of endocan in liver fibrosis was enhanced by the inclusion in the developed, prognostic model of other variables, such as TG, age, gender, and antihypertensive therapy, showing an excellent discriminatory capability (area under the curve (AUC) = 0.840) for advanced fibrosis, with sensitivity and specificity of 72.41% and 86.36%, respectively, vs. the poor discriminatory capability of endocan for liver fibrosis as a single marker (AUC = 0.667). On the contrary, serum endocan levels did not differ between patients with chronic hepatitis B (n = 55) and chronic hepatitis C (n = 19) with no/mild fibrosis and severe fibrosis in another study [39] that included a smaller number of patients than the previous one (n = 147) [9].

## 6. Endocan and Type 2 Diabetes Mellitus

### 6.1. Circulating Endocan Levels in Type 2 Diabetes Mellitus

In a former observational study enrolling 59 patients with prediabetes, 102 patients with T2DM, and 117 controls, it was shown that serum endocan levels were significantly higher in the T2DM cohort compared to the prediabetes and control groups [7]. Notably, multivariate logistic ordinal regression analysis revealed that an increase in serum endocan levels by one unit resulted in a two-fold increase in the odds of higher HbA1c levels [7], suggesting an interconnection between this novel biomarker of endothelial dysfunction with poor glycemic control in T2DM. Of note, in a more recently published case-control study recruiting 23 patients with T2DM and 23 healthy controls, it was shown that plasma endocan levels were significantly lower in T2DM patients compared to controls, while they remained unchanged after hyper-insulinemic euglycemic clamp, a finding suggestive of an absence of a relationship between circulating endocan levels and glycemic variability [43]. However, according to previous data retrieved from a proteomics and miRNA profiling analysis in a total of 1157 subjects without baseline T2DM from the Relationship between Insulin Sensitivity and Cardiovascular disease (RISC) cohort, it has been documented that endocan might even be a prognostic marker of the decline in β-cell function and the development of impaired glucose tolerance and T2DM [44].

### 6.2. Relationship between Endocan, Endothelial Dysfunction, and Subclinical Atherosclerosis in Type 2 Diabetes Mellitus

A formerly published case-control study enrolling 88 patients with T2DM and 88 healthy controls showed that circulating endocan levels are higher among subjects with T2DM and concomitant endothelial dysfunction compared to those with baseline T2DM without endothelial dysfunction, documenting that endocan is an independent predictor of endothelial dysfunction, significantly increasing the corresponding odds by almost 46% [45]. In another case-control study enrolling 69 patients with T2DM, with and without subclinical atherosclerosis, and 28 healthy controls, it was shown that: (1) circulating levels of endocan were significantly higher among subjects with T2DM, (2) endocan levels positively correlated with HbA1c levels, (3) endocan levels positively correlated with increased cIMT levels, and (4) endocan levels represented an independent risk factor for the presence of subclinical atherosclerosis, increasing the corresponding risk almost by 98% [46]. According to another recently published case-control study enrolling 42 subjects with prediabetes and 42 healthy controls, circulating endocan levels might be lower among those participants with prediabetes [28]. However, endocan was significantly correlated with increased cIMT, a finding suggesting that it might be predictive of subclinical atherosclerosis even at the prediabetes stage [28].

### 6.3. Endocan and Microvascular Complications of Type 2 Diabetes Mellitus

Data retrieved from recently published observational studies are suggestive of a significant relationship between endocan levels and microvascular complications of T2DM. In a former case-control study enrolling 53 healthy controls, 46 patients with T2DM and peripheral diabetic neuropathy (DN), and 53 patients with T2DM without DN, it was shown that: (1) patients with T2DM and DN had significantly higher circulating endocan levels compared to healthy controls, and (2) patients with T2DM and DN also had significantly higher circulating endocan levels than subjects without underlying DN, suggesting an emerging role of endocan in several mechanisms implicated into DN pathogenesis, including endothelial dysfunction, angiogenesis, and inflammation [47]. Of note, subjects with DN and concomitant insulin treatment had significantly higher endocan levels compared to those treated with oral antidiabetic drug classes [47].

In another observational study enrolling 137 patients with T2DM and normal baseline renal function, categorized according to the albuminuria level, it was shown that the urine albumin–creatinine ratio (UACR) had a significant negative bivariate correlation with serum endocan levels, while patients with macroalbuminuria had lower circulating endocan levels, compared to those with normoalbuminuria and microalbuminuria [48]. Therefore, serum endocan might represent an additional prognostic factor for diabetic nephropathy among subjects with T2DM [48].

Finally, there is also some evidence suggesting a potential role of endocan in diabetic retinopathy (DR) pathogenesis. In a case-control study enrolling 44 patients with T2DM proliferative DR and 29 subjects without underlying T2DM, analysis of vitreous fluid samples revealed that endocan levels were significantly higher in patients with DR compared to non-diabetic subjects [49]. In addition, a significant correlation between endocan levels in vitreous fluid and levels of angiogenic markers, such as VEGF and soluble vascular endothelial-cadherin (sVE-cadherin), was shown, suggesting the potential role of endocan in neo-angiogenesis occurring in DR subjects [49]. Of importance, significant expression of endocan in vascular endothelial cells and myofibroblasts isolated from epiretinal membranes of the enrolled participants was documented [49], reflecting the crucial role of endocan expression in endothelial cell activation and angiogenesis in DR. Similar results were reported in another observational study, enrolling 30 subjects with T2DM and cataract, 30 subjects with T2DM and DR and cataract, 30 subjects with cataract but without T2DM, and 30 healthy controls, showing significantly increased levels of endocan in blood and aqueous humor in subjects with T2DM, DR, and concomitant cataract, compared to the other groups, a finding suggestive of its role in the pathogenesis of DR in subjects with T2DM [50].

### 6.4. Endocan and Macrovascular Complications of Type 2 Diabetes Mellitus

There is only one study to date assessing the potential role of endocan in subjects with T2DM developing macrovascular complications. Specifically, in a former cross-sectional study from China, enrolling 72 patients with T2DM, of whom 38 had a recent history of acute myocardial infarction while the rest had no CVD, and 33 normotensive controls, it was demonstrated that: (1) subjects with underlying T2DM had significantly higher serum endocan levels, (2) subjects with T2DM and recent myocardial infarction had significantly higher serum endocan levels, compared to subjects with T2DM and no history of CVD, and (3) in patients with T2DM, serum endocan positively correlated with markers of inflammation, such as hsCRP and the neutrophil-to-lymphocyte ratio; however, no significant correlation with age was shown [51]. Therefore, endocan may be a novel biomarker for the early prediction of cardiovascular complications of T2DM, representing underlying endothelial dysfunction and affected by residual, low-grade inflammation [51].

Concerning another cardiovascular complication of T2DM, namely diabetic cardiomyopathy, endothelial dysfunction appears to be a significant contributing pathophysiologic mechanism (but not the only one), mainly mediating the restrictive phenotype (heart failure with preserved left ventricular ejection fraction, HFpEF) [52,53]. Unfortunately, to date, there are no studies assessing whether endocan is implicated in diabetic cardiomyopathy pathogenesis and its prognostic implications concerning disease progression and related complications [1].

## 7. Endocan and Arterial Hypertension

### 7.1. Circulating Endocan Levels in Arterial Hypertension

In a former cross-sectional study enrolling 18 subjects with primary arterial hypertension (HTN) and 23 matched, normotensive controls, it was shown that serum endocan levels were significantly higher in the HTN group, and showed significant, positive correlation with markers of subclinical atherosclerosis (such as cIMT) and markers of inflammation (such as hsCRP) [54]. Similar results were shown by another cross-sectional study enrolling 61 patients with newly diagnosed HTN and 30 healthy controls, confirming a positive, significant correlation between endocan levels and both cIMT and systolic blood pressure [55]. Another cross-sectional study enrolling 67 patients newly diagnosed with essential HTN and 70 healthy controls also documented that endocan levels were significantly higher among hypertensive subjects, and were positively correlated with aortic elastic properties assessed by echocardiography, namely aortic strain and aortic distensibility, also confirming the strong relationship between endocan levels and target-organ damage in HTN [56]. Of course, some contradictory data also show no association between circulating endocan levels and target-organ damage in a large cohort of 132 asymptomatic hypertensive subjects [57].

In a cross-sectional analysis recruiting 104 patients with HTN and 21 healthy controls, it was confirmed that plasma endocan levels were significantly higher among subjects with primary HTN, compared to controls [18]. A significant, negative correlation between endocan and BMI and leukocyte count was also shown [18]. Similar results were demonstrated by an even larger cross-sectional analysis of 938 normotensive, pre-hypertensive, and hypertensive subjects, showing significantly increased levels of circulating endocan concentration among patients with established HTN, indicative of endothelial dysfunction and target-organ damage [58]. In a previous cross-sectional study enrolling 90 patients with HTN and 44 normotensive subjects, it was shown that circulating endocan levels were significantly higher among hypertensive subjects, compared to normotensive controls, while an increase in endocan levels by 1 pg/mL was associated with an increase in the odds of the presence of HTN by 32.2% [11].

Of note, in a recent cross-sectional analysis, with 28 patients with primary aldosteronism (PA), a major cause of secondary HTN, 14 patients with primary HTN, and 28 healthy controls, it was demonstrated that circulating endocan levels were significantly lower in the PA group, compared to the other groups [59]. However, no other studies to date have assessed the correlation between endocan and secondary HTN.

Last, but not least, another observational study enrolling 35 dipper and 35 non-dipper hypertensive subjects, along with 35 normotensive controls, documented that circulating endocan levels were significantly higher in the non-dipping group, compared to dippers and healthy controls [60]. Additionally, multivariate logistic regression analysis showed that endocan is an independent, significant predictor of non-dipping status [60]. Despite being preliminary, these findings might suggest the crucial role of endocan as a prognostic marker of early target-organ damage in patients with HTN.

### 7.2. Relationship between Endocan Levels and Atherosclerotic Cardiovascular Disease in Subjects with Hypertension

A former cross-sectional study, enrolling 190 hypertensive subjects assessed for possible CAD by undergoing coronary angiography, demonstrated that hypertensive patients with confirmed CAD had significantly higher circulating endocan levels [61]. Endocan was shown to be an independent predictor for the presence of CAD, by resulting in a two-fold significant increase in the odds for CAD presence [61]. A similar study enrolling 164 hypertensive subjects and 55 controls also confirmed that patients with concomitant HTN and CAD had significantly higher endocan levels, compared to those patients having only background HTN [62]. Endocan was also shown to be an independent predictor of CAD, with a similar increase in the corresponding odds [62].

## 8. Endocan and Atherosclerotic Cardiovascular Disease

### 8.1. Circulating Endocan Levels in Coronary Artery Disease

As previously mentioned, endocan has been proposed as a novel, prognostic marker for ASCVD [1].

Among individuals with a history of a recent ST-segment elevation myocardial infarction (STEMI), high serum endocan levels have been proven to be an independent predictor of incomplete ST-segment resolution (STR), representing a marker of insufficient myocardial perfusion after primary percutaneous coronary intervention, and thus, of worse clinical prognosis [63]. Similar results were provided by another relevant study enrolling patients with STEMI undergoing primary percutaneous coronary intervention, showing that higher circulating endocan levels were an independent predictor of the occurrence of the no-reflow phenomenon and impaired myocardial perfusion [64].

In another prospective cross-sectional analysis of subjects with a recent acute coronary syndrome (ACS), it was demonstrated that serum endocan levels were an independent predictor of recurrent major adverse cardiovascular events (MACEs) after hospital discharge, and thus of significantly worse prognosis [65].

### 8.2. Circulating Endocan Levels and Cerebrovascular Disease

In another cross-sectional study recruiting patients with a recent acute ischemic stroke, it was documented that higher endocan levels were associated with a significant increase in the odds for a composite endpoint of death or major disability within the first months after the occurrence of the stroke, despite that after adjustment for confounding factors, the increase in the odds of death was non-significant [66]. It has also been shown that endocan levels were higher among individuals with large artery atherosclerotic stroke, compared to controls [67]. Endocan might also be significantly associated with worse outcomes within the first week of hospitalization due to acute ischemic stroke, demonstrating a significant, short-term prognostic value [67].

### 8.3. Circulating Endocan Levels and Heart Failure

Even in the field of heart failure (HF), endocan has emerged as a novel, prognostic biomarker, with an increase in circulating endocan levels by 1 ng/mL being associated with a significant increase in the risk of HF-related mortality or hospitalization requiring inotropic support by 47%, after adjustment for several confounding factors among individuals with chronic, stable HF [68].

Previous studies have confirmed that endocan is an independent predictor of different forms of ASCVD, even among patients with an established disease [1,2]. Therefore, close monitoring of circulating endocan levels may be predictive of surrogate outcomes, including mortality and cardiovascular morbidity [1,2].

## 9. Endocan and Renal Disease

### Circulating Endocan Levels and Chronic Kidney Disease

An early cross-sectional study enrolling 251 patients with chronic kidney disease (CKD), stages 1–5, and 60 controls published almost a decade ago suggested that: (1) circulating endocan levels are significantly higher among subjects with CKD compared to the controls, (2) endocan levels are progressively higher across advanced stages of CKD, and (3) the circulating endocan levels among subjects with CKD are significantly correlated with inflammatory markers and indices of endothelial dysfunction, suggesting that it might be an important predictor of all-cause death among this specific population [17]. Similar results were obtained from another, recently published observational study which enrolled 30 patients with CKD stage 5 under hemodialysis, 30 patients with CKD not receiving hemodialysis, and 30 controls, which confirmed a strong relationship between endocan levels and the stage of CKD [69]. The interconnection between endocan levels, systemic inflammation, subclinical atherosclerosis, and endothelial dysfunction was also confirmed [69].

Among subjects with end-stage CKD receiving peritoneal dialysis, serum endocan levels were significantly correlated with patients’ nutritional status, overall survival, and incidence of MACEs [70]. However, no association between endocan and the risk for hospital admission within one year was demonstrated [70].

In the field of diabetic nephropathy, as discussed previously, only two observational studies have addressed the relationship of this microvascular complication with endocan levels. In the first study, it was demonstrated that among individuals with underlying T2DM, circulating endocan levels were significantly higher among those with microalbuminuria or macroalbuminuria, compared to those with normoalbuminuria [8], documenting that endocan might be a novel marker for the progression of diabetic nephropathy. However, the opposite results were demonstrated by the second study, also enrolling subjects with T2DM [48], in which the researchers demonstrated that patients with macroalbuminuria had lower circulating endocan levels, compared with those having normoalbuminuria, and showing a negative correlation between endocan levels and UACR. To date, those two studies are the only available studies addressing this important topic, unfortunately with conflicting results.

A brief summary of endocan levels in different cardiometabolic disorders is presented in Table 2.

## 10. The Impact of Cardiometabolic-Targeted Therapies on Endocan Levels

Unfortunately, the data concerning the impact of cardiometabolic-targeted therapies on circulating endocan levels are scarce. In the field of hypertension, a former randomized trial enrolling 37 subjects with newly diagnosed essential hypertension demonstrated that 3-month treatment either with valsartan or amlodipine resulted in a significant reduction in circulating endocan levels, leading to a greater numeric decrease with amlodipine [71]. Of note, no correlation between the change in blood pressure and circulating endocan levels was shown [71]. In the field of ASCVD, a former randomized trial recruiting 63 patients with a recent acute myocardial infarction undergoing percutaneous coronary intervention, who were assigned either to high-intensity atorvastatin or rosuvastatin treatment, demonstrated that after a 4-week treatment, rosuvastatin, but not atorvastatin, resulted in a significant reduction in circulating endocan levels [72]. However, a numeric reduction was documented with both statins [72].

As far as patients with T2DM are concerned, a previously published observational study enrolling 77 subjects with insufficient glycemic control (baseline glycated hemoglobin levels: 10.7% ± 2.28%) revealed that optimization of medical treatment and attainment of the desired glycemic control resulted in a significant reduction in circulating endocan levels after a 3-month follow-up [73]. Unfortunately, because the study was conducted and published before 2015, the subjects did not receive any of the currently available antidiabetic drug classes with proven cardio-renal benefits, namely sodium-glucose co-transporter-2 (SGLT-2) inhibitors and glucagon-like peptide-1 (GLP-1) receptor agonists [73]. In addition, the researchers did not perform any further sub-analysis on the change in endocan levels, according to the administered antidiabetic drug classes [73]. There are only some experimental data suggesting that metformin can enhance endocan transcription and circulating protein levels in human umbilical vein endothelial cells (HUVECs) exposed to acute hyperglycemia [74]. However, no human studies exist to date.

Therefore, there is a significant gap in the current knowledge concerning the true impact of newer, cardiometabolic-targeted treatment options on circulating endocan levels, and of course whether that impact could mediate some of the observed cardiovascular benefits seen with classes such as SGLT-2 inhibitors, GLP-1 receptor agonists, proprotein convertase subtilisin/kexin type 9 (PCSK9) inhibitors, and mineralocorticoid receptor antagonists.

## 11. Future Perspectives

Discovered two decades ago, endocan still represents an intriguing biomarker related to inflammation. Endocan is investigated in many cardiometabolic diseases that are tightly connected with inflammation, such as obesity, polycystic ovary syndrome, metabolic syndrome, non-alcoholic fatty liver disease, type 2 diabetes mellitus, hypertension, atherosclerotic cardiovascular disease, kidney disease, etc. Although controversial results have been demonstrated, the discrepancies might be attributed to the different examined ethnic groups, different age and gender distribution, the phenotypes of adiposity, the duration and the extent of obesity, and the sample size of the studied population. Longitudinal, multicentric studies with large sample sizes are needed to further examine the role of endocan in cardiometabolic disorders.

Since endocan has emerged as a novel endothelial dysfunction marker, the discovery of potential therapeutic strategies for patients with increased cardiometabolic burden would be of great importance to postpone and/or prevent the onset and progression of ASCVD.

## Figures and Tables

**Table 1:** Molecular actions of endocan.

Endothelial Cells	Leukocytes	Vascular Smooth-Muscle Cells	Tumor Cells
↑ VEGF, ↑ permeability of blood vessels [1,2]	↑ Modulation of leukocytes’ migration [2,5]	↑ Vascular smooth-muscle cell proliferation [1,2,5]	↑ Cell proliferation [1,2,5]
↑ Upregulation of cell adhesion molecules’ expression (↑ VCAM-1, ↑ ICAM-1, ↑ E-Selectin) [1,2]	↑ Leukocytes’ adhesion and activation [2,5]	↑ Neointima formation ↑atherogenesis [1,2,5]	↑ Tumor development and neo-angiogenesis [3,5]

Legend: ↑ increased; VEGF—vascular endothelial growth factor; VCAM-1—vascular cell adhesion molecule-1; ICAM-1—intercellular adhesion molecule.

**Table 2:** Endocan levels in different cardiometabolic disorders.

Endocan Level (Serum/Plasma)	Cardiometabolic Disorder	Sample Size	Reference
↑ positive correlation with BMI, WC, WHtR, BRI, BAI, VAI, LAP, and CMI	Obesity	n = 177 adults free of diabetes, CVD, and CKD	Klisic et al. [6]
↑	Obesity and gestational diabetes mellitus	n = 40 women with obesity and gestational diabetes mellitus vs. n = 10 pregnant women with normal glucose tolerance	Murthi et al. [20]
↑ positive correlation with cIMT	Obesity	n = 80 children with obesity vs. n = 80 healthy controls	Nalbantoğlu et al. [23]
↓ negative correlation with CRP	Obesity	n = 70 overweight/obese postmenopausal women	Janke et al. [16]
↑ positive correlation with HDL-c level, but an inverse association with BMI and CRP	PCOS	n = 88 women with PCOS vs. n = 87 age- and BMI-matched controls n = 54 normal weight PCOS women vs. n = 34 overweight/obese PCOS women	Delibas et al. [19]
↑ positive correlation with BMI, WC, hs-CRP, HOMA-IR, FAI, cIMT	PCOS	n = 80 women with PCOS vs. n = 80 controls	Bicer et al. [10]
↑	MetS	n = 34 adults with MetS vs. n = 56 MetS-free	Iwańczyk et al. [26]
↑	MetS	n = 30 children with MetS vs. n = 30 healthy children	Halici et al. [27]
↑	Prediabetes	n = 59 adults with prediabetes vs. n = 117 controls	Klisic et al. [7]
↓	Prediabetes	n = 42 adults with prediabetes vs. n = 42 controls	Arman et al. [28]
↓	MetS	n = 44 adults with MetS vs. n = 26 controls	Boyuk et al. [29]
↑ positive correlation with TG, glucose, and HbA1c; negative correlation with HDL-c	T2DM	n = 42 adults with T2DM vs. n = 64 diabetes-free controls	Klisic et al. [30]
↑ positive correlation with other endothelial factors (ICAM-1, VCAM-1, VEGF), and inflammation factors (IL-6), remnant lipoprotein cholesterol, and the atherogenic index of plasma	Postprandial lipemia	n = 54 hyperlipidemic adults vs. n = 28 normolipidemic controls	Ozer Yaman et al. [31]
↑	NAFLD (determined by ultrasonography) and CAD (determined by coronary angiography)	n = 66 adults with NAFLD and CAD vs. n = 11 adults with NAFLD and CAD-free	Elsheikh et al. [37]
↑ positive correlation with BMI, WC, glucose, HbA1c, TG, ALT, GGT, and hsCRP; negative correlation with HDL-c	NAFLD (determined by FLI)	n = 147 adults with NAFLD (FLI ≥ 60) vs. n = 64 (FLI < 30)	Klisic et al. [9]
↑	NAFLD (determined by ultrasonography and/or a liver biopsy)	n = 37 adults with NAFLD with T2DM and n = 19 adults with NAFLD but T2DM-free vs. n = 25 healthy controls	Dallio et al. [38]
↓	NAFLD (determined by liver biopsy)	n = 38 adults with NAFLD vs. n = 34 controls	Tok et al. [39]
↓ negative correlation with BMI	NAFLD (determined by ultrasonography) and MetS	n = 40 adults with MetS and NAFLD vs. n = 20 healthy controls	Erman et al. [40]
↔	NAFLD (determined by ultrasonography) and obesity	n = 40 obese children/adolescents with and without NAFLD (n = 60) vs. n = 40 controls	Ustyol et al. [41]
↔	NAFLD (determined by ultrasonography) and obesity	n = 26 overweight/obese children/adolescents with NAFLD vs. n = 48 overweight/obese children/adolescents without NAFLD	Bălănescu et al. [42]
↑ positive correlation with glucose and HbA1c; negative correlation with TG and ALT	Liver fibrosis (determined by BARD score)	n = 124 adults with advanced fibrosis vs. n = 23 with no/mild fibrosis	Klisic et al. [9]
↔	Liver fibrosis (determined by liver biopsy)	n = 55 adults with chronic hepatitis B and n = 19 adults with chronic hepatitis C with no/mild fibrosis vs. severe fibrosis	Tok et al. [39]
↑	T2DM	n = 102 adults with T2DM vs. n = 59 controls	Klisic et al. [7]
↓	T2DM	n = 23 adults with T2DM vs. n = 23 healthy controls	Moin et al. [43]
↑ positive correlation with cITM and 24 h urine protein excretion level	T2DM	n = 88 adults with T2DM with and without endothelial dysfunction vs. n = 88 healthy controls	Balamir et al. [45]
↑ positive correlation with glucose, HbA1c, and cIMT	T2DM with subclinical atherosclerosis	n = 69 adults with T2DM with (n = 27) and without (n = 42) subclinical atherosclerosis vs. n = 28 healthy controls	Lv et al. [46]
↑	T2DM and DN	n = 46 adults with T2DM with DN and n = 53 adults with T2DM without DN vs. n = 53 healthy controls	Bilir et al. [47]
↓ negative correlation with urine albumin–creatinine ratio	T2DM and diabetic nephropathy	n = 137 adults with T2DM with macroalbuminuria (n = 35) had lower circulating endocan levels vs. T2DM with normoalbuminuria (n = 55) and microalbuminuria (n = 47)	Cikrikcioglu et al. [48]
↑	T2DM and DR	n = 44 adults with T2DM and DR vs. n = 29 diabetes-free controls	Abu El-Asrar et al. [49]
↑ positive correlation with hsCRP and neutrophil-to-lymphocyte ratio	T2DM and AMI	n = 38 adults with T2DM and AMI and n = 34 adults with T2DM with no cardiovascular disease vs. n = 33 normotensive controls	Qiu et al. [51]
↑	HTN	n = 90 adults with HTN vs. n = 44 healthy controls	Klisic et al. [11]
↑ positive correlation with hsCRP and cIMT	HTN	n = 18 adults with HTN vs. n = 23 normotensive controls	Oktar et al. [54]
↑ positive correlation with cIMT	HTN	n = 61 adults with HTN vs. n = 30 healthy controls	Balta et al. [55]
↑ negative correlation with aortic distensibility and aortic strain	HTN	n = 67 adults with HTN vs. n = 70 healthy controls	Çelik et al. [56]
↑ negative correlation with BMI and leukocyte count	HTN	n = 104 adults with HTN vs. n = 21 healthy controls	Musialowska et al. [18]
↑	HTN	n = 938 adults, i.e., normotensive (n = 291), pre-hypertensive (n = 268), and hypertensive (n = 379) adults	Turgunova et al. [58]
↑	HTN and CAD	n = 164 adults with HTN with CAD (n = 72) and without CAD (n = 92) vs. n = 55 controls having only background HTN	Wang et al. [62]
↑ progressively higher across advanced stages of CKD positive correlation with cIMT, hsCRP, and diabetes	CKD	n = 251 adults with CKD stages 1–5 vs. n= 60 controls	Yilmaz et al. [17]
↑ positive correlation with cIMT	CKD	n = 30 adults with CKD stage 5 under hemodialysis, n = 30 adults with CKD not receiving hemodialysis vs. n= 30 controls	El-Senosy et al. [69]
↑	T2DM and diabetic nephropathy	n = 56 adults with nephropathy (microalbuminuria or macroalbuminuria) vs. n = 40 adults with normoalbuminuria and n = 35 healthy non-diabetic controls	Ekiz-Bilir et al. [8]

Legend: ↑ higher level, ↓ lower level, ↔ no difference. BMI—body mass index; WC—waist circumference; WHtR—waist-to-height ratio; BRI—body roundness index; BAI—body adiposity index; VAI—visceral adiposity index; LAP—lipid accumulation product; CMI—cardiometabolic index; CVD—cardiovascular disease; CKD—chronic kidney disease; cIMT—carotid intima-media thickness; hsCRP—high-sensitivity C-reactive protein; HOMA-IR—homeostasis model assessment of insulin resistance; FAI—free androgen index; PCOS—polycystic ovary syndrome; HDL-c—high-density lipoprotein cholesterol; TG—triglycerides; MetS—metabolic syndrome; HbA1c—glycated hemoglobin; ICAM-1—intercellular adhesion molecule; VCAM-1—vascular cell adhesion molecule-1; VEGF—vascular endothelial growth factor; IL-6—interleukin-6; NAFLD—non-alcoholic fatty liver disease; ALT—alanine aminotransferase; GGT—gamma glutamyl transferase; FLI—fatty liver index; CAD—coronary artery disease; T2DM—type 2 diabetes mellitus; DN—diabetic neuropathy; DR—diabetic retinopathy; AMI—acute myocardial infarction; HTN—hypertension.
